# Supplementary material for: The Conserved Colletotrichum spp. Effector Candidate CEC3 Induces Nuclear Expansion and Cell Death in Plants
Source: Front Microbiol. 2021 Sep 3;12:682155. doi: 10.3389/fmicb.2021.682155 (PMC8446390; doi:10.3389/fmicb.2021.682155)
Supplement: Supplementary file 2 [file Data_Sheet_1.DOCX]

Supplementary Material

# Agrobacterium-mediated transformation of *Colletotrichum* fungi

1. Pre-culture
2. Dilute 50 ul of agrobacterium stock in 5 ml of LB containing 50 µg/ml kanamycin, 25 µg/ml carbenicillin, and 50 µg/ml rifampicin in a 50 ml falcon tube. Incubate at 28˚C overnight with shaking at 220 rpm in the dark.

Note: We use *Agrobacterium tumefaciens* strain AGL1 to transform *Colletotrichum* fungi using this protocol.

Note: Carbenicillin and rifampicin are used to select *A. tumefaciens* strain AGL1. Kanamycin is used to select bacteria containing a binary vector.

1. Next morning, collect *Agrobacterium* cells by centrifuging at 4,000 x*g* for 10 min at room temperature.
2. Discard the supernatant and suspend the agrobacterium pellet using 5 ml of fresh Induction Medium (IM; see below). Centrifuge at 4,000 x*g* for 10 min at room temperature.
3. Discard the supernatant. Add 5 ml of fresh IM and check the *Agrobacterium* concentration. Dilute *Agrobacterium* to OD_600_ = 0.6 in 2.0 ml of IM containing 200 µM acetosyringone and 50 µg/ml kanamycin in a 14 ml falcon tube.
4. Incubate for 6-8 h at 25˚C with shaking at 250 rpm in the dark.
5. Prepare and dilute fungal spore suspension
6. Make spore suspension from a sporulated plate with 0.05% Tween20.
7. Check spore concentration using a hemocytometer.
8. Dilute the suspension with 80% glycerol to give a final concentration of 15% glycerol and 10^7^-10^8^ spore/ml. Store 100 μl aliquots at -80˚C.
9. Thaw the frozen aliquot at room temperature and make serial dilutions before use.

Note: We usually prepare serial dilutions from 10^5^ spore/ml to 10^3^ spore/ml. The minimum requirement is 250 μl of 10^5^ spore/ml suspension assuming three dilutions (200 μl each of 10^5^, 10^4^, 10^3^ spore/ml).

1. Co-culture
2. Prepare CCM plates containing 200 µM acetosyringone and 50 µg/ml kanamycin.

Note: Use freshly prepared plates.

1. Carefully place 9 cm diameter Hybond -N+ membranes on CCM plates without air bubbles.
2. Mix 200 μl of *Agrobacterium* preculture with 200 μl fungal spore suspension.
3. Spread 200 μl of the culture onto each of the CCM plates covered with the Hybond -N+ membranes using sterile glass beads.
4. Seal CCM plates using insulating tape and incubate at 25-28˚C for 24-48 h in the dark.

Note: We usually incubate at 25˚C for 48 h.

1. Selection
2. Transfer the Hybond -N+ membranes from CCM plates to selection plates.
3. Seal selection plates using insulating tape and incubate at 25-28˚C for 5-7 days.
4. Cut about 1-2 mm^2^ square of the Hybond -N+ membrane containing a single colony using a sterile scalpel. Transfer the membrane piece to a new selection plate.
5. Incubate selection plates at 25˚C in the dark for a few days. After fungal colonies grow, extract genomic DNA from mycelial samples and perform genotyping.
6. Transfer the positive colonies to PDA plates and use them for experiments.

**Appendix**

1. Preparation of *Agrobacterium* stocks
   1. Spread *Agrobacterium* cells harboring a binary vector on an LB plate.
   2. Incubate plates at 28˚C for 1-2 days in the dark.
   3. Suspend *Agrobacterium* cells in 25 ml sterile water.
2. Centrifuge at 4,000 x*g* for 10 min at room temperature, then discard the supernatant.
   1. Resuspend the cells in 25 ml sterile water.
   2. Measure OD_600_.
3. Centrifuge at 4,000 x*g* for 10 min at room temperature, then discard the supernatant.
   1. Suspend cells in 15% glycerol to a final OD_600_ = 10.
   2. Make 50 μl of aliquots and store at -80˚C.
4. Preparation of media and reagents

- **Stock solutions**
- 10×MM (PN): 100 mM K_2_HPO_4_ (8.71 g/500 ml), 100 mM KH_2_PO_4_ (6.81 g/500 ml),40 mM (NH_4_)_2_SO_4_ (2.64 g/500 ml)
- 10×MM (metal): 25 mM NaCl (0.73 g/500 ml), 20 mM MgSO_4_ 7H_2_O (2.47 g/500 ml), 7 mM CaCl_2_·2H_2_O (0.51 g/500 ml), 90 μM FeSO_4_·7H_2_O (12.5 mg/500 ml), filter-sterilized
- 40% glucose, autoclaved
- 80% (v/v) glycerol, autoclaved
- AS stock: 0.2 M acetosyringone in DMSO, filter-sterilized
- Km stock: 50 mg/ml kanamycin in water, filter-sterilized
- Hyg stock: 50 mg/ml hygromycin B in water, filter-sterilized
- Car stock: 50 mg/ml carbenicillin in water, filter-sterilized
- Rif stock: 100 mg/ml rifampicin in DMSO, filter-sterilized
- Cefo stock: 300 mg/ml cefotaxime in water, filter-sterilized
- **Minimal medium (MM)**: 10 mM K_2_HPO_4_, 10 mM KH_2_PO_4_, 4 mM (NH_4_)2SO_4_, 2.5 mM NaCl, 2 mM MgSO_4_, 0.7 mM CaCl_2_, 9 μM FeSO_4_, 10 mM glucose

1. Mix 20 ml of 10×MM (PN) and 159.1 ml of water, then autoclave.
2. After autoclave, add 20 ml of 10×MM (metal) and 0.9 ml of 40% glucose.

Note: MM alone is not required for this protocol.

- **Induction medium (IM)**: MM (10 mM glucose), 40 mM MES, 0.5% glycerol, pH 5.3

1. Mix 20 ml of 10×MM (PN) 20 ml, 158 ml of water, and MES 1.71 g.
2. Adjust pH to 5.3 with HCl and autoclave.
3. After autoclave, add 20 ml of 10×MM (metal), 0.9 ml of 40% glucose, 1.25 ml of 80% glycerol.

- **Co-cultivation medium (CCM)**: MM (5 mM glucose), 40 mM MES, 0.5% glycerol, pH 5.3

1. Mix 40 ml of 10×MM (PN), 316 ml of water, and MES 3.41 g.
2. Adjust pH to 5.3 with HCl and add 6 g of Bacto agar, then autoclave.
3. After autoclave, add 40 ml of 10×MM (metal), 0.9 ml of 40% glucose, 2.5 ml of 80% glycerol, 400 μl of Km stock, and 400 μl of AS stock.
4. Pour the medium to sterilized Petri dishes.

- **Selection plates**

1. Mix PDA powder mix and water, then autoclave.
2. After autoclaving, add 40% glucose, Hyg stock, and Cefo stock to a final concentration of 0.6 M glucose, 100 µg/ml hygromycin B, and 100 µg/ml cefotaxime.

Note: Cefotaxime is used to select against *Agrobacterium*.

1. Pour the medium to sterilized 9 cm diameter Petri dishes.
